# A Novel Expanding Mechanism of Gastrointestinal Microrobot: Design, Analysis and Optimization

**DOI:** 10.3390/mi10110724

**Published:** 2019-10-26

**Authors:** Wei Wang, Guozheng Yan, Zhiwu Wang, Pingping Jiang, Yicun Meng, Fanji Chen, Rongrong Xue

**Affiliations:** 1 School of electronic information and electrical engineering, Shanghai Jiao Tong University, Shanghai 200240, China; aries-wang@sjtu.edu.cn (W.W.); zwwang@sjtu.edu.cn (Z.W.); jpp99@sjtu.edu.cn (P.J.); yicun.meng@sjtu.edu.cn (Y.M.); cfj0717@sjtu.edu.cn (F.C.); rongrongxue@sjtu.edu.cn (R.X.); 2 Institute of Medical Robotics, Shanghai Jiao Tong University, Shanghai 200240, China

**Keywords:** gastrointestinal microrobot, expanding mechanism, elastodynamics, finite element analysis, structure optimization

## Abstract

In order to make the gastrointestinal microrobot (GMR) expand and anchor in the gastrointestinal tract reliably, a novel expanding mechanism of the GMR is proposed in this paper. The overlapping expanding arm is designed to be used to increase the variable diameter ratio (ratio of fully expanded diameter to fully folded diameter) to 3.3, which makes the robot more adaptable to the intestinal tract of different sections of the human body. The double-layer structure of the expanding arm increases the contact area with the intestine, reducing the risk of intestinal damage. The kinematics and mechanical model of the expanding arm are established, and the rigid velocity, rigid acceleration, and expanding force of the expanding arm are analyzed. The elastodynamics model of the expanding arm is established. Through the finite element analysis (FEA), the velocity, acceleration, and the value and distribution of the stress of the expanding arm under elastic deformation are obtained. Based on the elastodynamics analysis, the structure of the expanding arm is optimized. By the structure optimization, the thickness of the expanding mechanism is reduced by 0.4mm, the weight is reduced by 31%, and the stress distribution is more uniform. Through the mechanical test, the minimum expanding force of the expanding mechanism is 1.3 N and the maximum expanding force is 6.5 N. Finally, the robot is tested in the rigid pipeline and the isolated intestine to verify the reliability and safety of the expanding mechanism.

## 1. Introduction

The morbidity and mortality of gastrointestinal malignancy and related diseases are increasing year by year, which has seriously threatened people’s health and even life [1,2]. Traditional endoscopy has some problems, such as missed detection, the painful process, and potential to cause serious complications. Capsule endoscopy can only rely on the passive movement of intestinal peristalsis and cannot carry out fixed-point detection of the gastrointestinal tract. It also does not have the function of expanding the intestinal tract and cannot be examined at the intestinal fold [3]. Therefore, the research focused on the gastrointestinal microrobot which can move autonomically and expand the intestinal tract is important [4,5,6,7,8].

The expanding mechanism is the key device for the gastrointestinal microrobot (GMR) to achieve active locomotion and anchoring in the intestinal tract. The performance of the GMR is directly related to the gastrointestinal tract safety, applicability and work efficiency [9]. The robot must have enough safety, so as to avoid damage to the intestine. In addition, due to different size of the intestine in different sections of the human body, a larger variable diameter ratio (VDR) can enable the robot to better diagnose the intestinal tract of different sections of the human body. Considering the actual operation conditions, in order to minimize the operation time, raising the locomotion efficiency of the robot is very important. The driven type of the expanding mechanism mainly includes the magnetic driven type [10,11,12,13,14] and the micromotor drive type [15,16,17,18,19]. The magnetically driven type expanding mechanism adopts an off-board power supply, which greatly reduces the power required by the on-board load. However, there are two problems with this type of mechanism. One is that it moves too fast, which makes it easy to cause impact on the intestinal tract. The other is that in practical application, real-time detection of robot posture is needed to apply the magnetic field in the corresponding direction, the technology is relatively complex. The advantage of the micromotor driving mode is that the action time is controllable, and there is no need to detect the robot posture, but the micromotor driving mode increases the power consumption of the board. Various institutions have developed different structural forms of expanding mechanism. The expanding mechanism designed by Chen W.W. et al. adopts the design of Archimedes’ spiral leg [20,21]. Its expanding force increases with the increase of expanding radius, which conforms to the mechanical properties of the intestine, but the tip of its expanding arm is too sharp, and it is easy to get stuck in the intestinal tract when rotating, which is easy to cause damage to the intestinal tract. The airbag expanding mechanism [22,23] has a high level of safety for the gastrointestinal tract, but it takes too long to inflate and deflate, and its working mode is inefficient, which is not suitable for practical operation. The leg expanding mechanism designed by He S., Gao J.Y. et al. [24,25,26] takes into account the problems of movement efficiency and safety, but its VDR is relatively small (only 2.56), which is not suitable for the diagnosis of intestinal tract with a large diameter, and its expansion tension is small. The expanding mechanism developed by Zhou H. et al. [27] has a simple structure, but the VDR and the expanding force are small. To sum up, the main difficulties in the design of the expanding mechanism at present lie in the aspect of VDR, expanding force, and work efficiency.

In this paper, a novel expanding mechanism of the inchworm-like GMR is designed, which adopts a double-layer overlapping design and has a VDR of 3.3. The average time for the expanding mechanism to complete an expanding or folding action is about 1.2 s, which has a high working efficiency. At present, the research of expanding mechanism mainly focuses on the analysis of expanding force, anchoring force, and kinematics when the mechanism is regarded as a rigid body, and there are few studies on the dynamics of the expanding arm when it is deformed under load. In order to further study the correlation performance in the actual situation, it is necessary to analyze the elastodynamics of the expanding mechanism. Due to the relatively narrow intestinal tract of the human body, the size of the robot is strictly limited. Since the lighter and smaller the robot, the more flexible and locomotion efficient the mechanism in the intestinal tract, it is necessary to optimize the structure of the mechanism to make the robot lightweight and miniaturized.

## 2. Robot System

### 2.1. Structure of the Mechanism

**Figure 1:**
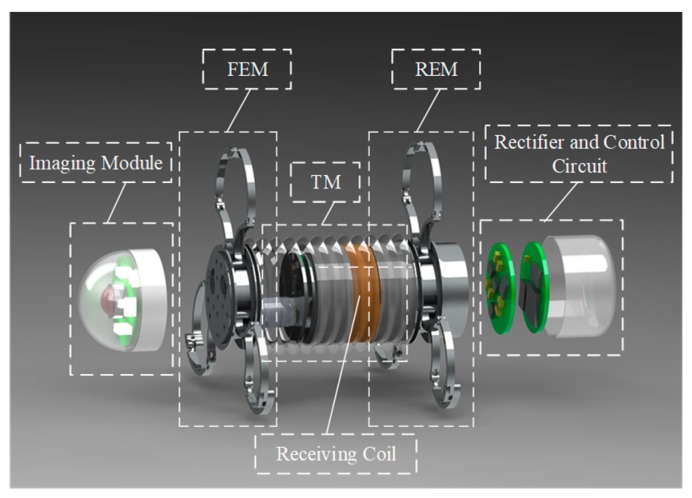
The structure of robot system.

The GMR system is mainly composed of four modules: the imaging module, the mechanical module, the wireless power receiving module, and the control circuit module, as shown in Figure 1. The imaging module is composed of a glass cover, a lens, LED light sources, a CMOS image sensor, and an image data wireless transmission circuit. The mechanical module is mainly composed of two expanding mechanisms and one telescoping mechanism and they are mainly made of titanium alloy. The wireless power receiving module is mainly composed of a receiving coil and a rectifying circuit.

The mechanical structure of the robot mainly consists of three parts: the front expanding mechanism (FEM), the rear expanding mechanism (REM), and the telescoping mechanism (TM). The FEM and REM have the same structure, the FEM moves relative to the TM, and the REM is fixed to the TM. The expanding mechanism is mainly composed of a driving device, a transmission device and three sets of expanding arms. The driving device is composed of a hollow cup motor and a reducer. The reducer adopts a 5-layer planetary gear type structure, and the reduction ratio of each layer is 4. The transmission device is mainly composed of three sets of external meshing gears. There are three sets of expansion arms, each consisting of four curved connecting rods.

**Figure 2:**
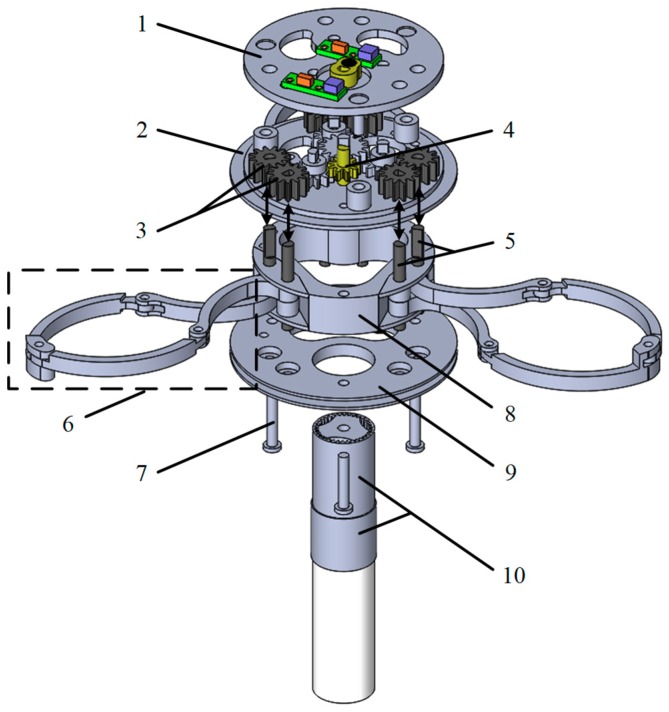
Structure of the expanding mechanism.

The structure of the expanding mechanism of GMR is shown in Figure 2. The main component names: (1) Top panel, (2) Middle panel, (3) Gear pair of expanding arm, (4) Drive gear, (5) Transmission shaft of expanding arm, (6) Expanding arm, (7) Bolt, (8) Pedestal, (9) Bottom panel, (10) Motor and reducer.

The motor provides driving force, the torque is reduced by the reducer, the force is transmitted to the transmission gear set, and then the transmission shaft is driven to rotate by the transmission gear set, thereby driving the expanding arm to achieve expanding and folding. The expanding arm is in contact with the intestinal tract during the expansion process. The larger the expanding radius is, the greater the resistance will be, resulting in the larger the current of the control circuit. It is assumed that: (1) the mechanical properties of various parts of the human digestive tract are the same, (2) the motor performance remains stable all the time. The upper limit of current is set in the control circuit through in vitro intestinal experiment. Once the upper limit of the current is exceeded, the expanding arm will stop expanding, so as to avoid damage to the intestinal tract caused by excessive expanding force.

The relevant parameters of the expanding mechanism are shown in Table 1.

### 2.2. Locomotion Principle

**Figure 3:**
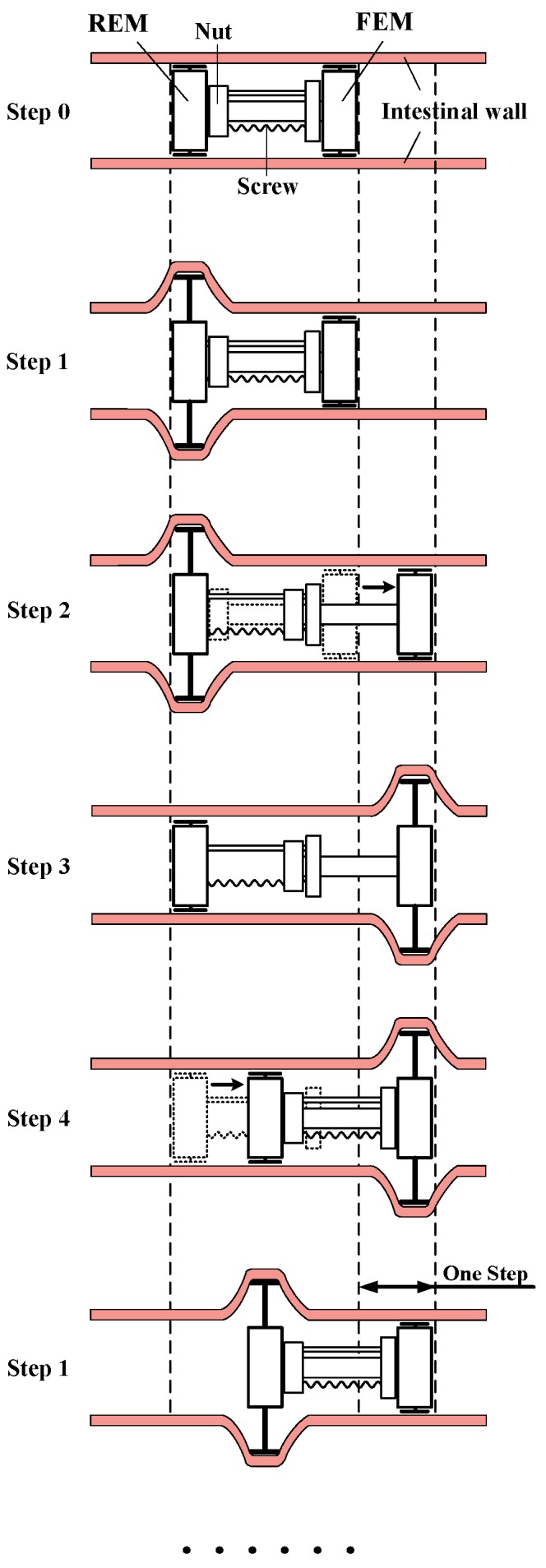
Locomotion principle of the inchworm-like GMR.

The locomotion principle is shown in Figure 3. Two expanding mechanisms of the GMR alternately expand and fold. The screw-nut pair of the telescoping mechanism drive the folded expanding mechanism to move, thereby the robot moves in the intestinal tract. Step 0: The FEM and REM are folded. Step 1: The REM expands. Step 2: The screw-nut pair drive the FEM to move forward. Step 3: The FEM expands first, and then the REM folds. Step 4: The screw-nut pair drive the REM to move forward. The inchworm-like GMR repeats the Step 1–4, thus moves forward.

## 3. Kinematics and Mechanics Analysis of the Expanding Arm

### 3.1. Kinematics Analysis

**Figure 4:**
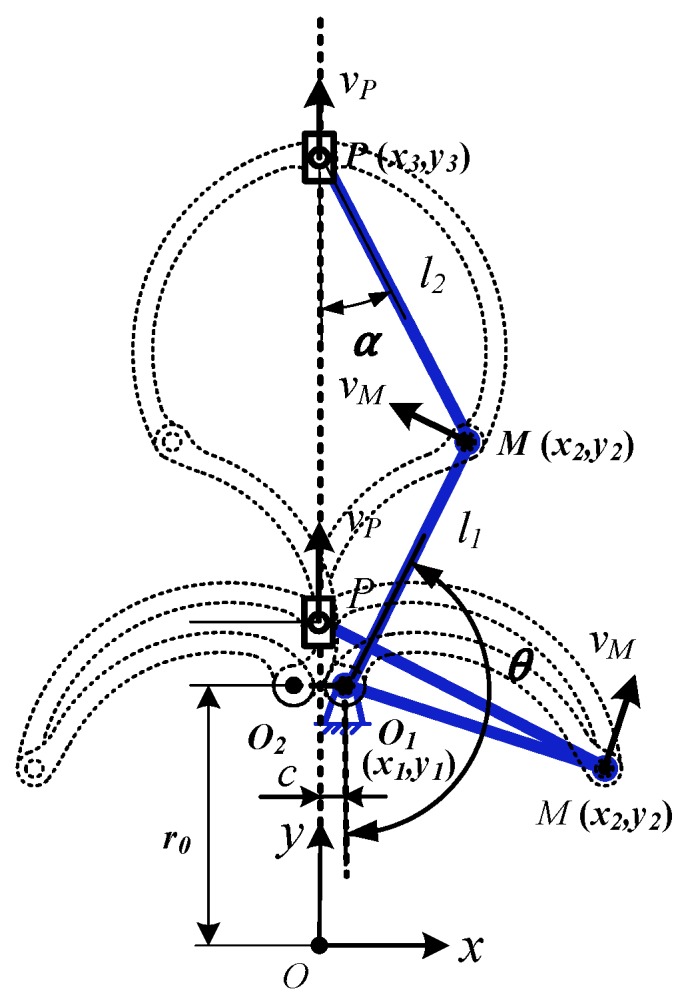
Kinematics analysis of the expanding arm.

For rigid body kinematics analysis of the expanding arm, the expanding arm is simplified into a slider-crank mechanism module, as shown in Figure 4. Create a Cartesian coordinate system *Oxy* with the center of the mechanism O as the origin, and define the coordinates of the hinge points *O***_1_**(*x*_1_,*y*_1_), *M*(*x*_2_,*y*_2_), *P*(*x*_3_,*y*_3_).

The mathematical model of the hinge point coordinate matrix ***S*** is as follows:

(1) $$S=\left[\begin{array}{cc} x_{1} & y_{1}\\ x_{2} & y_{2}\\ x_{3} & y_{3} \end{array}\right]$$

The constraint equation is:

(2) $$\left\{ \begin{array}{l} x_{2}-x_{1}{=l}_{1}\cdot \sin\theta \\ y_{2}-y_{1}=-l_{1}\cdot \cos\theta \\ x_{3}-x_{2}=-l_{2}\cdot \sin\alpha \\ y_{3}-y_{2}{=l}_{2}\cdot \cos\alpha \\ l_{1}\cdot \sin\theta +x_{1}{=l}_{2}\cdot \sin\alpha \end{array}\right.$$

where, *l*_1_, *l*_2_ are the lengths of the rod *O*_1_*M* and the rod *MP*, respectively; *θ*, *α* are the angles of the rod *O*_1_*M* and the rod *MP* with the vertical direction, respectively. 

The point *O_1_* is a fixed point, let *x*_1_ = *c*, *y*_1_ = *r*_0_, and *c*, *r*_0_ is constant. Then in the *Oxy* coordinate system, the mathematical model of each hinge point coordinate matrix is:

(3) $$S=\left[\begin{array}{cc} x_{1} & y_{1}\\ x_{2} & y_{2}\\ x_{3} & y_{3} \end{array}\right]=\left[\begin{array}{cc} c & r_{0}\\ l_{1}\cdot \sin\theta +c & -l_{1}\cdot \cos\theta +r_{0}\\ 0 & Q-l_{1}\cdot \cos\theta +r_{0} \end{array}\right]$$

where, $Q=\sqrt{l_{2}^{2}-{\left(l_{1}\cdot \sin\theta +c\right)}^{2}}$.

The first and second derivatives of coordinate matrix ***S*** with respect to time t are velocity matrix V and acceleration matrix A of each hinge point respectively.

(4) $$V=\left[\begin{array}{cc} {\dot{x}}_{1} & {\dot{y}}_{1}\\ {\dot{x}}_{2} & {\dot{y}}_{2}\\ {\dot{x}}_{3} & {\dot{y}}_{3} \end{array}\right]=\omega \left[\begin{array}{cc} 0 & 0\\ l_{1}\cdot \cos\theta & l_{1}\cdot \sin\theta \\ 0 & l_{1}\cdot \sin\theta -l_{1}\cdot \cos\theta \cdot \frac{l_{1}\cdot \sin\theta +c}{Q} \end{array}\right]$$

(5) $$A=\left[\begin{array}{cc} {\ddot{x}}_{1} & {\ddot{y}}_{1}\\ {\ddot{x}}_{2} & {\ddot{y}}_{2}\\ {\ddot{x}}_{3} & {\ddot{y}}_{3} \end{array}\right]=\omega^{2}\left[\begin{array}{cc} 0 & 0\\ -l_{1}\cdot \sin\theta & l_{1}\cdot \cos\theta \\ 0 & l_{1}\cdot \cos\theta +l_{1}\sin\theta \cdot \frac{l_{1}\cdot \sin\theta +c}{Q}-\frac{l_{1}^{2}\cdot l_{2}^{2}\cdot \cos^{2}\theta }{Q^{3}} \end{array}\right]$$

It is the angular velocity of the rod *O*_1_*M*. 

According to the above Equations (1)–(5), the expanding radius *r_c_* and the velocity and acceleration of point *P* can be obtained. As follows:

(6) $$\left\{ \begin{array}{l} r_{c}=y_{3}\\ v_{P}=\sqrt{{\dot{x}}_{3}^{2}+{\dot{y}}_{3}^{2}}\\ a_{P}=\sqrt{{\ddot{x}}_{3}^{2}+{\ddot{y}}_{3}^{2}} \end{array}\right.$$

The relevant design parameters of the expanding mechanism are shown in Table 2.

**Figure 5:**
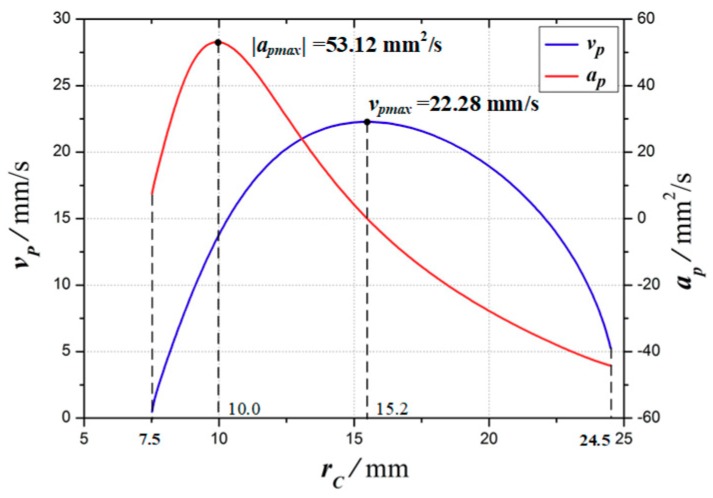
Theoretical velocity and acceleration of point *P*.

At different expanding radii *r_c_* (7.5 mm ≤ *r_c_* ≤ 24.5 mm), the velocity *v_p_* and acceleration *a_p_* of point *P* are shown in Figure 5. When the expanding radius *r_c_* = 10.0 mm, the absolute value of acceleration at point *P* reaches the maximum, |*a_p_*|_max_ = 53.12 mm^2^/s. When the expanding radius *r_c_* = 15.2 mm, the point *P* velocity reaches the maximum, *v_p_*_max_ = 22.28 mm/s.

### 3.2. Expanding Force Analysis

**Figure 6:**
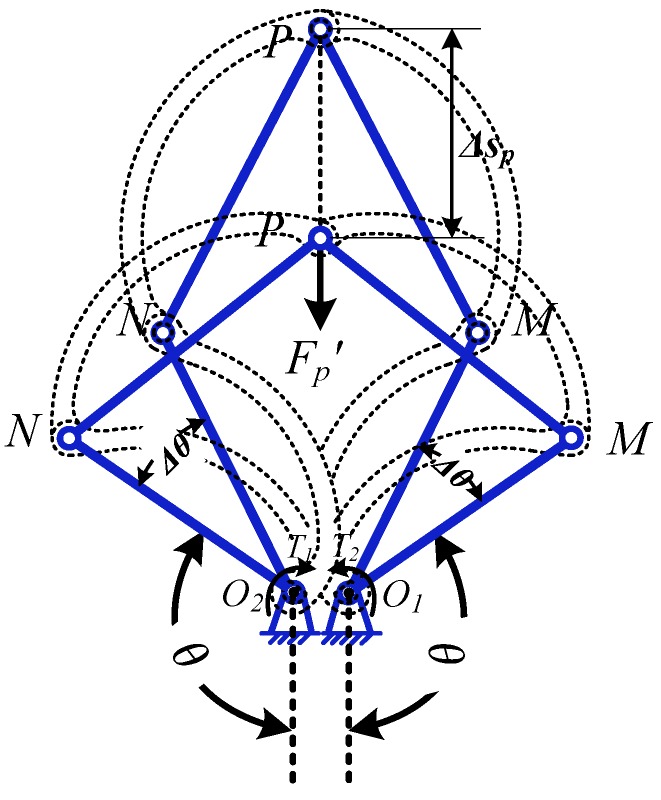
Mechanical analysis of the expanding arm.

For expanding force analysis of the expanding mechanism, the expanding arm can be considered as a static system, as shown in Figure 6.

According to the principle of virtual work, the virtual work done by the moments *T*_1_ and *T*_2_ on the small-angle increment *Δθ* is *T*_1_·*Δθ* and *T*_2_*·Δθ*, respectively.

(7) $$\left\{ \begin{array}{l} T_{1}\cdot \Delta \theta +T_{2}\cdot \Delta \theta -F_{P}^{\prime }\cdot \Delta s_{p}=0\\ T=T_{1}+T_{2} \end{array}\right.$$

From Equation (3):

(8) $$\Delta s_{p}=\left(l_{1}\cdot \sin\theta -l_{1}\cdot \cos\theta \frac{\left(l_{1}\cdot \sin\theta +c\right)}{\sqrt{l_{2}^{2}-{\left(l_{1}\cdot \sin\theta +c\right)}^{2}}}\right)\cdot \Delta \theta$$

According to Equations (7),(8), the expanding force *F_P_* can be derived:

(9) $$F_{P}=F_{P}^{\prime }=T\cdot {\left(l_{1}\cdot \sin\theta -l_{1}\cdot \cos\theta \frac{\left(l_{1}\cdot \sin\theta +c\right)}{\sqrt{l_{2}^{2}-{\left(l_{1}\cdot \sin\theta +c\right)}^{2}}}\right)}^{-1}$$

where, *r_c_* is the radius of expanding; $F_{P}$ and $F_{P}^{\prime }$ are the expanding force and reaction force at point *P*; *T*_1_ and *T*_2_ are the maximum driving torques acting on *O*_1_ and *O*_2_, respectively; *T* is the maximum driving torque of the expanding arm under the rated voltage of the motor, the value is tested to be 32 N·mm.

**Figure 7:**
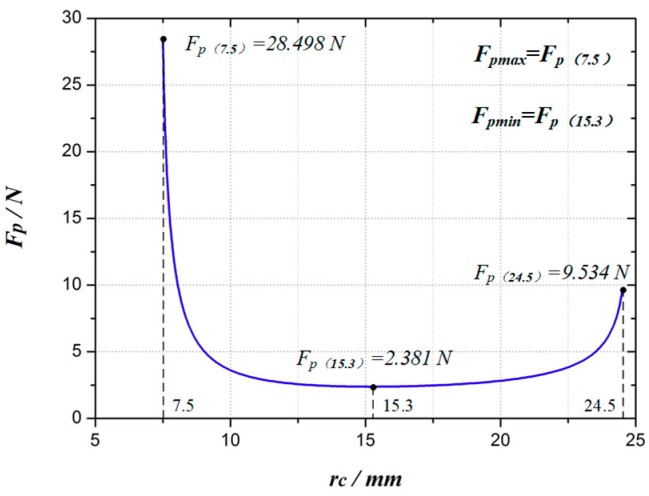
The curve of expanding force to expanding radius.

According to Equations (1) and (9), the relationship between the expanding force at point *P F_P_* and the expanding radius *r_c_* can be obtained, as shown in Figure 7. As the mechanism arm expands, the expanding force *F_P_* first gets smaller and then larger. When *r_c_* = 7.5 mm (the fully folded state of the expanding arm), *F_P_* is the maximum, *F_P_*_max_ = *F_P_*(7.5) = 28.498 N. After that, *F_P_* sharply decreases. *F_P_* changes relatively small when *r_c_* is from 10 mm to 22 mm. When *r_c_* = 15.3 mm, *F_P_* is the minimum, *F_P_*_min_ = *F_P_*(15.3) = 2.381 N. When *r_c_* = 24.5 mm (the fully expanded state of the expanding arm), *F_P_*(24.5) = 9.534 N.

## 4. Elastodynamic Analysis and Optimization

### 4.1. Elastodynamic Model

**Figure 8:**
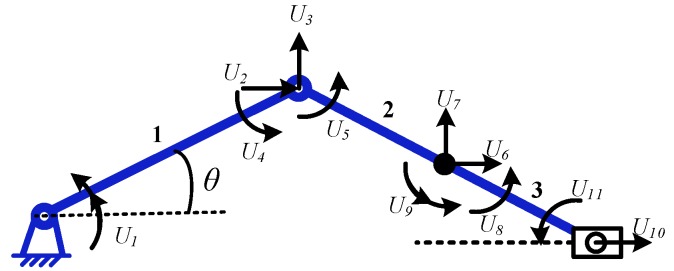
The model of the simplified expanding arm.

The expanding arm is simplified into a slider-crank mechanism module and divides the module into three units. Think of the crank as a unit–unit 1, the connecting rod is divided into two units—units 2 and 3. Set 11 generalized coordinates in the mechanism *U* = [*U*_1_ *U*_2_ … *U*_11_]^T^, as shown in Figure 8.

For each unit, define the angle between the beam element and the horizontal direction as *θ*. define the generalized coordinate array of the unit *u* = [*u*_1_ *u*_2_ … *u*_8_]^T^. where u_1_, u_5_ are the longitudinal displacements of the nodes at both ends of each unit; u_2_, u_6_ are the lateral displacements of the nodes at both ends of each unit; *u*_3_, *u*_7_ are the elastic corners of the nodes at both ends of each unit; *u*_4_, *u*_8_ are the curvatures of the nodes at both ends of each unit. The unit generalized coordinate array in the absolute coordinate system is defined as *U^e^*. For the *i*-th unit, their relationship is shown as follows:

(10) $$\left\{ \begin{array}{l} u_{i}=RU_{i}^{e}\\ U_{i}^{e}=B_{i}U \end{array}\right.\;(i=1,2,3)$$

where *R* is the coordinate transformation matrix; *B_i_* is the coordinate coordination matrix, which is an 8 × 11 matrix in this model, and the *R* and *B_i_* is as follows:

$$R=\left[\begin{array}{cc} \begin{array}{cccc} \cos\theta & \sin\theta & 0 & 0\\ -\sin\theta & \cos\theta & 0 & 0\\ 0 & 0 & 1 & 0\\ 0 & 0 & 0 & 1 \end{array} & 0_{4\times 4}\\ 0_{4\times 4} & \begin{array}{cccc} \cos\theta & \sin\theta & 0 & 0\\ -\sin\theta & \cos\theta & 0 & 0\\ 0 & 0 & 1 & 0\\ 0 & 0 & 0 & 1 \end{array} \end{array}\right]$$

$$B_{1}=\left[\begin{array}{c} 0_{\left(3\times 11\right)}\\ \begin{array}{cc} \begin{array}{cccc} 1 & 0 & 0 & 0\\ 0 & 1 & 0 & 0\\ 0 & 0 & 1 & 0\\ 0 & 0 & 0 & 1\\ 0 & 0 & 0 & 0 \end{array} & 0_{\left(5\times 7\right)} \end{array} \end{array}\right]$$

$$B_{2}=\left[\begin{array}{cc} 0_{\left(4\times 5\right)} & \begin{array}{cccccc} 1 & 0 & 0 & 0 & 0 & 0\\ 0 & 1 & 0 & 0 & 0 & 0\\ 0 & 0 & 1 & 0 & 0 & 0\\ 0 & 0 & 0 & 1 & 0 & 0 \end{array}\\ \begin{array}{ccccc} 0 & 1 & 0 & 0 & 0\\ 0 & 0 & 1 & 0 & 0\\ 0 & 0 & 0 & 0 & 1\\ 0 & 0 & 0 & 0 & 0 \end{array} & 0_{\left(4\times 6\right)} \end{array}\right]$$

$$B_{3}=\left[\begin{array}{cc} 0_{\left(4\times 9\right)} & \begin{array}{cc} 1 & 0\\ 0 & 0\\ 0 & 1\\ 0 & 0 \end{array}\\ \begin{array}{cc} 0_{\left(4\times 5\right)} & \begin{array}{cccc} 1 & 0 & 0 & 0\\ 0 & 1 & 0 & 0\\ 0 & 0 & 1 & 0\\ 0 & 0 & 0 & 1 \end{array} \end{array} & 0_{\left(4\times 2\right)} \end{array}\right]$$

The differential equation of motion of the mechanical system can be expressed as:

(11) $$M\ddot{U}+C\dot{U}+\mathrm{KU}=F-{M\ddot{U}}_{r}$$

Where, *M*, *C*, and *K* are the mass matrix, damping matrix, and stiffness matrix of the system, respectively. *F* is the generalized force array of the system, and ${\ddot{U}}_{r}$ is the rigid body acceleration array of the system, which can be obtained by the rigid body motion analysis of the mechanism. *U* is the system generalized coordinate array, $\dot{U}$ and $\ddot{U}$ are the first and second derivatives of the system generalized coordinate array *U* with respected to time *t*, respectively.

### 4.2. Elastodynamic Analysis by FEA

**Figure 9:**
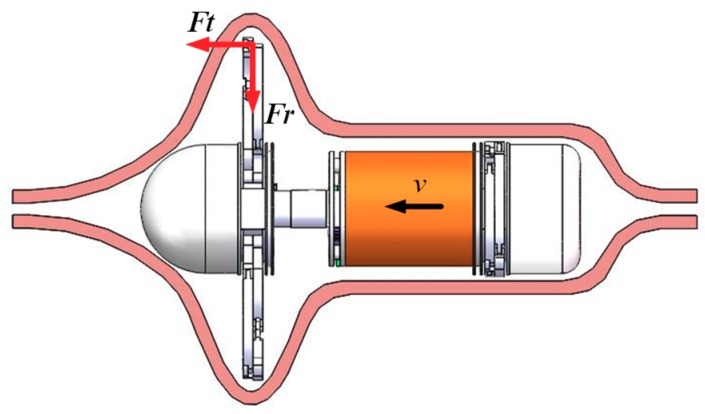
The force condition of GMR in the intestine.

During the locomotion of the robot in the intestine, the force exerted by the intestine on the expanding arm can be divided into two directions. One is the resistance *Fr* in the radial direction formed by the elastic deformation of the intestine, and the other is the resistance *Ft* of the intestine to the expanding arm in the direction of locomotion of the robot, as shown in Figure 9. According to the above expanding force analysis, the theoretical radial resistance *Fr* on the expanding arm can be obtained. According to literature [28], *Ft* ≤ 0.6 N.

Since the expanding arm is an arc structure, if it is equivalent to a straight beam with a uniform section, some errors will be caused to the result, so the elastodynamic analysis of the expanding arm is carried out by the FEA software ANSYS [29].

**Figure 10:**
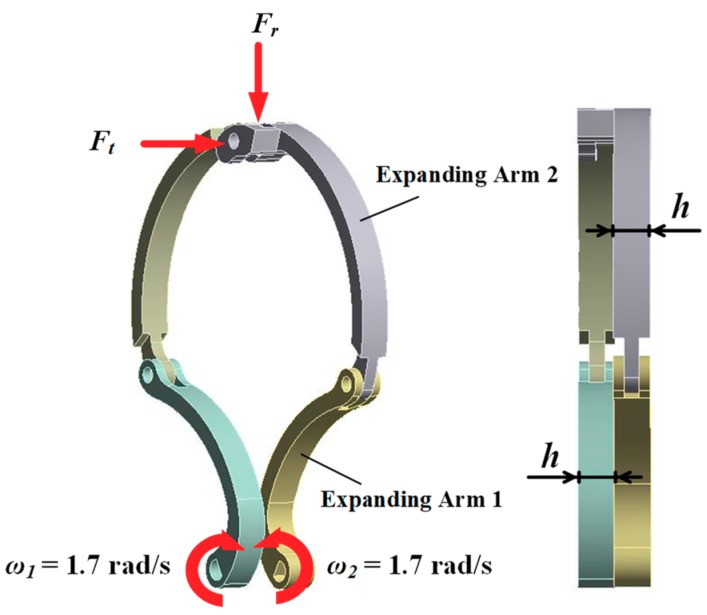
Load applied to the expanding arm.

The load applied to the expanding arm in ANSYS is shown in Figure 10. According to the above analysis, a radial resistance *Fr* and an axial resistance *Ft* = 0.6 N are applied to the expanding arm, and the velocity load *ω* = 1.7 rad/s is applied to the two D-shaped holes of the expanding arm respectively. Expanding mechanism design must meet the requirements of the thickness of two layer expanding arm + the thickness of bottom plate (0.8 mm) + the thickness of two layer backing plate (2 mm × 0.4 mm) ≤ the thickness of reducer (6 mm) − half of the thickness of connection sleeve (0.5 mm × 4 mm), so the thickness of monolayer expanding arm ≤ 1.2 mm. In order to ensure the strength, the maximum thickness of the monolayer expanding arm should be 1.2 mm before in-depth strength analysis.

**Figure 11:**
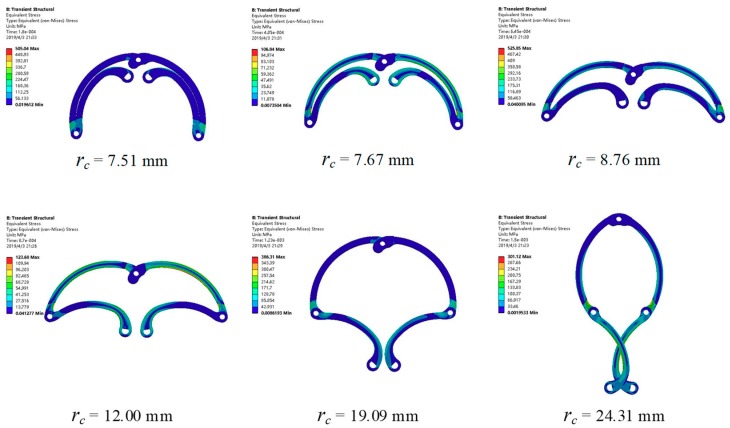
The equivalent stress of expanding arm.

**Figure 12:**
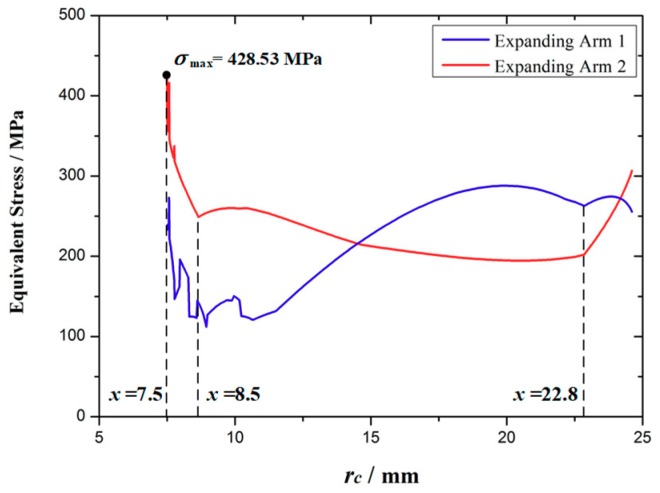
The curves of the equivalent stress.

The equivalent stress distribution of the expanding arm is shown in Figure 11. Figure 12 shows the maximum equivalent stress values of the expanding arms 1 and 2 with different expanding radii. It can be seen that with the expansion of the expanding arm, the maximum equivalent stress appears alternately on the expanding arm 1 and 2. The maximum equivalent stress of the whole motion process appears on expanding arm 2 in the initial expanding phase and the maximum value σ_max_ = 428.53 MPa. The material of the expanding arm is Ti-6Al-4V ELI, its yield strength σ_s_ = 950 MPa. The safety factor [s] is set to 1.1, so the safety stress of expanding arm is σ_[s]_ = σ_s_/[s] = 864 MPa. As σ_max_ < σ[s], the strength of expanding arm meets requirements.

**Figure 13:**
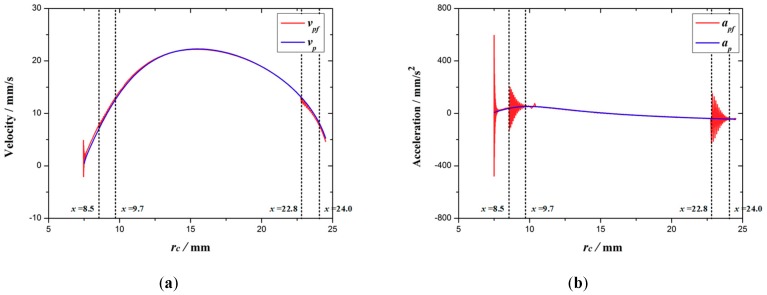
The response curves of velocity (**a**) and acceleration (**b**).

Based on the FEA, the response of the velocity *v_pf_* and the acceleration *a_pf_* of point *P* is compared with the theoretical value *v_p_* and *a_p_* as shown in Figure 13a,b. As can be seen from the figure, the velocity and acceleration of the expanding arm fluctuate greatly in three stages, namely *r_c_* = 7.5 mm stage, *r_c_* = 8.5–9.7 mm stage, and *r_c_* = 22.8–24.0 mm stage. The corresponding maximum deviation values of velocity |*Δv*|_max_ and acceleration |*Δa*|_max_ are shown in Table 3. From the comparison between Figure 12 and Figure 13, it can be seen that when the stress mutation occurs in the expanding arm, the velocity and acceleration of point *P* will fluctuate.

### 4.3. Structure Optimization of the Expanding Arm

As can be seen from the above FEA, the maximum stress of the expanding arm is far less than the safe stress, and the stress is mainly concentrated at the joint of each arm and the edge of the expanding arm, so the structure of the expanding arm needs to be optimized.

The thickness of the expanding arm is first optimized. The maximum equivalent stress is less than the safe value as the boundary condition. The thickness of the expanding arm is used as the optimization variable. The objective of the optimization is to minimize the thickness of the expansion arm. As shown in the following formula:

(12) $$\left\{ \begin{array}{l} h_{i}=1.2,1,0.8\;\cdots \;\mathrm{mm}\;(i=1,2,3\cdots )\\ \sigma_{\max-h}\leq \sigma_{\left[s\right]}\\ \min(h) \end{array}\right.$$

where *h_i_* is the thickness of the expanding arm, *σ*_max-*h*_ is the maximum equivalent stress of the expanding arm with different thicknesses. The FEA is carried out on the expanding arms with different thickness respectively to obtain the maximum equivalent stress in the whole movement process, as shown in Table 4. It can be seen from Table 4 that when the thickness of the expanding arm is 0.8 mm, the stress of the expanding arm does not meet the safety requirement, so the thickness of the expanding arm is optimal when it is 1 mm.

**Figure 14:**
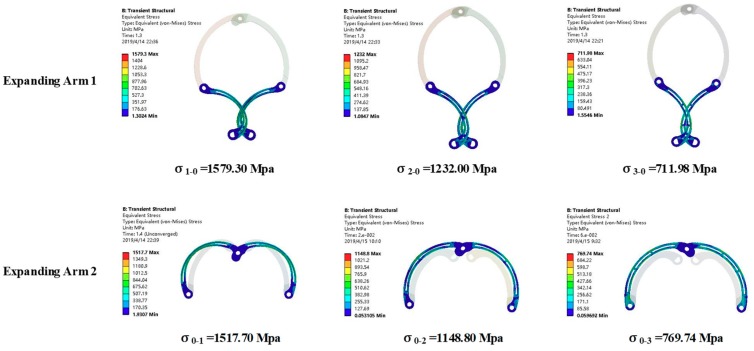
Optimization process of the expanding arm.

It can be seen from the stress distribution of the expanding arm that the stress in the middle region is much less than the stress in the edge region. The structure optimization of expanding arm 1 and 2 is carried out respectively. First, hollow out the middle region of the expanding arm. Then, the middle region of the expansion arm is divided into segments 1, 2, 3... until the maximum equivalent stress of the expanding arm is less than the safety stress. The optimization process is shown in Figure 14. From the analysis results, it can be concluded that the stress of the hollow part of expanding arm 1 and 2 meets the safety requirements when they are divided into three segments.

**Figure 15:**
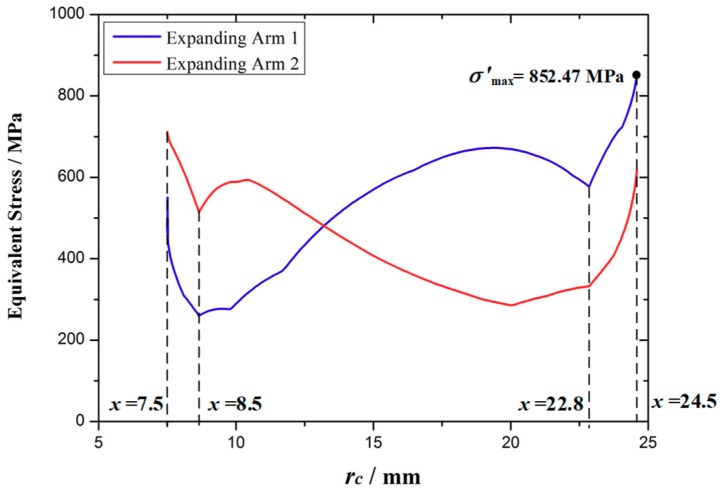
The equivalent stress of the optimized expanding arm.

Figure 15 shows the equivalent stress curve of the optimized expanding arm 1 and 2 during the movement. It can be seen that the stress of the optimized expanding arm is increased, and the overall maximum equivalent stress appears on the expanding arm 1, the maximum value σ'_max_ = 852.47 MPa < σ_[s]_ = 864 MPa, it meets the requirements of safety strength.

**Figure 16:**
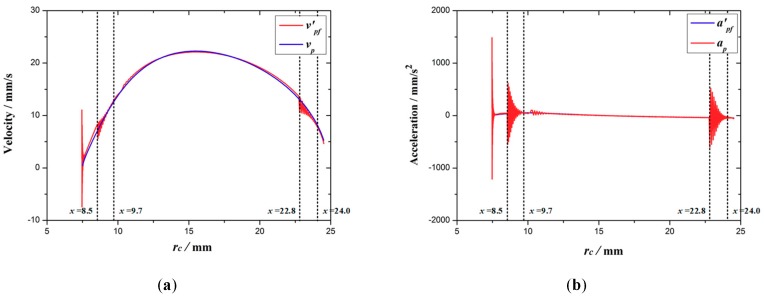
The response curves of velocity (**a**) and acceleration (**b**) after optimization.

The comparison between the velocity *v'_pf_* and acceleration *a'_pf_* at point *P* of the optimized expanding arm and the theoretical value is shown in Figure 16a,b. As before structural optimization, the velocity and acceleration of the optimized expanding arm fluctuate greatly in three stages. The corresponding maximum deviation values of velocity |*Δv*|max, and acceleration |*Δa*|max are shown in Table 5. Affected by the increase of stress, the fluctuation of velocity and acceleration at point *P* of expanding arm also increases.

**Figure 17:**
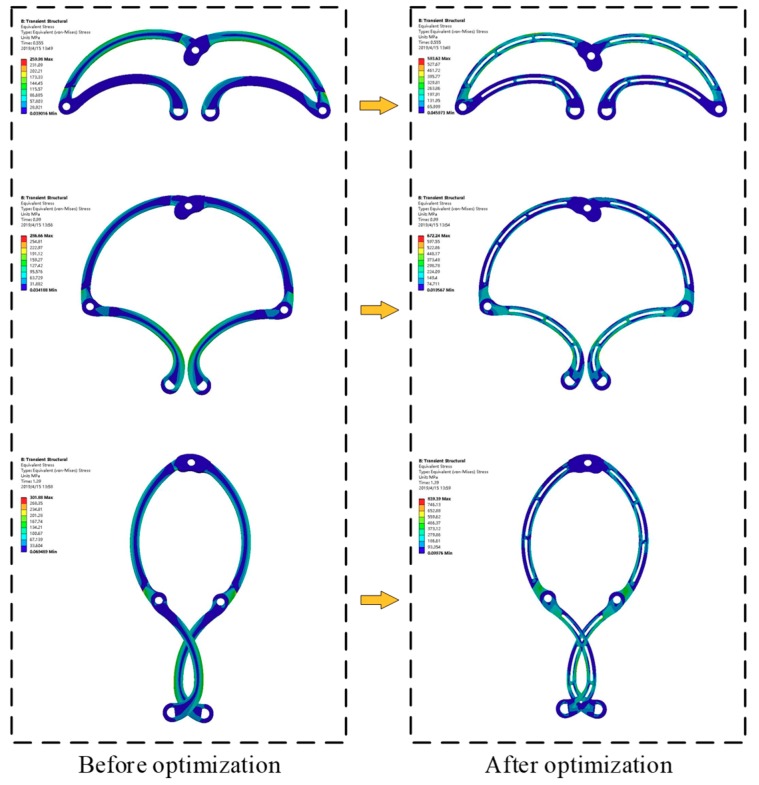
Comparison of stress distribution before and after optimization.

Figure 17 shows the comparison of equivalent stress with different expanding radii before and after the expansion arm optimization. After optimization, the overall thickness of the expanding arm is reduced by 0.4 mm, the weight of expanding arm is reduced by 31%.

## 5. Experiment

### 5.1. Expanding Force Test

**Figure 18:**
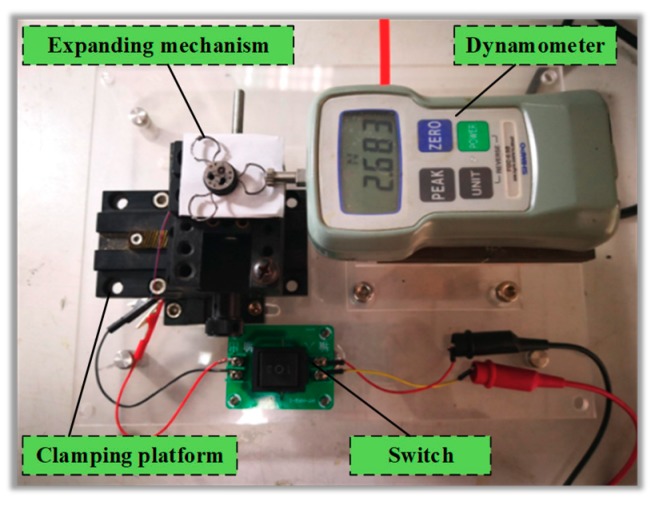
Mechanical performance test platform.

According to theoretical analysis, the expanding force is large in the fully folded stage, which may cause intestinal damage. Therefore, a mechanical test platform is built to test the actual expanding force of the expanding mechanism, as shown in Figure 18. The test platform consists of the DC power supply, dynamometer, control switch, clamping device, and base. The expanding mechanism is fixed on the clamping device, and the knob on the clamping device is used to adjust the distance between the expanding mechanism and the probe of the dynamometer, so as to measure the expanding force under different expanding radius. The control switch is used to control the forward and reverse of the motor and to protect the motor.

**Figure 19:**
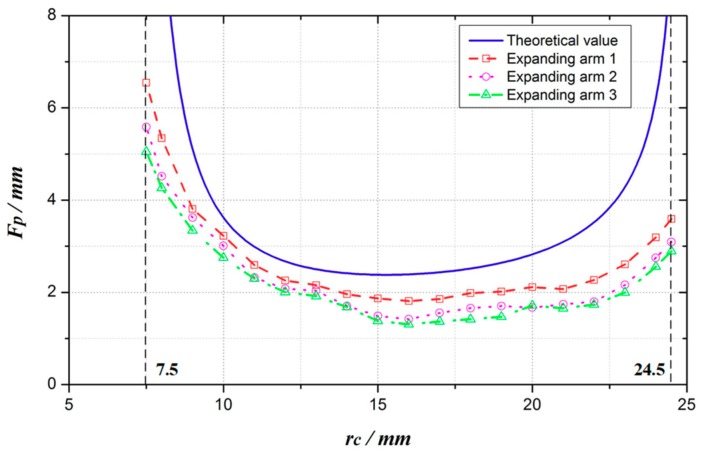
Experimental and theoretical value of expanding force.

Based on this platform, the expanding forces of the expanding arms 1, 2 and 3 are tested at the interval of 1 mm and the limit positions of 7.5 mm and 24.5 mm respectively. Each group of expanding arms is tested three times, and the average value is obtained. The experimental and theoretical curves of the expanding forces of the three groups of expanding arms are compared, as shown in Figure 19.

The analysis of the experimental results is as follows:

The three groups of experimental curves have the same trend as the theoretical curves, and the experimental values are less than the theoretical values. This is due to the efficiency of gear transmission and friction loss between mechanisms.

The maximum experimental value of three groups of expanding arms is 6.5 N, and the minimum experimental value is 1.3 N. The expanding force of expanding arm 1 is larger than that of expanding arm 2 and 3. In the process of installation, the running-in degree and transmission gear of expanding arm 1 is better than that of expanding arm 2 and 3, and the friction resistance is smaller in the course of motion, so that the expanding force is larger than that of the other two groups.

When the expanding radius is from 7.5 mm to 10 mm and from 22 mm to 24.5 mm, the slope of the curve is larger so that the small disturbance will cause a huge error to the expanding force, so the error between the experimental value and the theoretical value is larger. When the expanding radius is 10 mm to 22 mm, the curve is relatively stable and the error is relatively small. The average experimental value of the expansion arm 1 is 79.30% of the theoretical value, the expansion arm 2 is 68.04%, and the expansion arm 3 is 63.90%.

### 5.2. Locomotion Experiment

**Figure 20:**
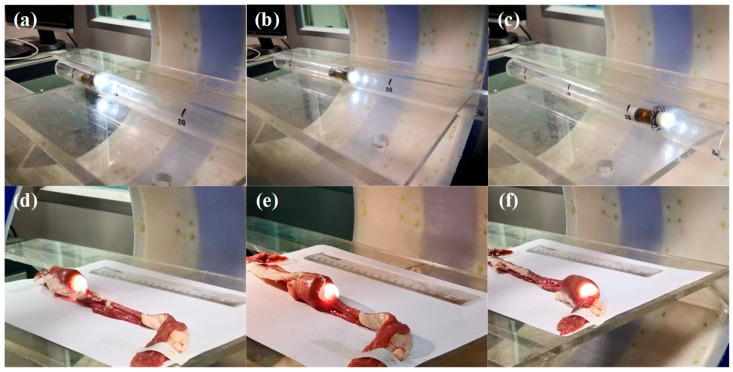
Robot locomotion experiment in the rigid pipe (**a**–**c**) and isolated intestine (**d**–**f**).

In order to verify the reliability and safety of the optimized mechanism, the robot is placed in a rigid tube (acrylic tube) and an isolated intestine (pig small intestine) for the locomotion experiment, as shown in Figure 20a–f. Figure 20a–c show the robot moving in the rigid pipe, and Figure 20d–f show the robot moving in the in vitro intestinal tract. During the locomotion of the robot, the mechanism works stably and reliably, and there is no obvious bending deformation of the expanding arm and no obvious damage to the intestine by a visual check. The average velocity of the robot in rigid tubes is 1.21 mm/s, while that in in vitro intestines was 0.94 mm/s. The velocity of the robot in the isolated intestine was significantly lower than that in the rigid tube. This is because the robot will lose step when it locomotes in the intestine. The main reasons for step loss are as follows:

The intestinal tract is malleable. As the robot locomotes in the intestine, the intestine will be elongated.

Compared with rigid pipes, the intestinal tract is relatively slippery. During the locomotion of the robot, there is slippage between the expanding mechanism and the intestinal tract, leading to the loss of step distance and the decrease of velocity.

## 6. Conclusion

In this paper, a novel GMR is proposed. Compared with other commercialized capsule endoscopy systems, the GMR proposed in this paper has the following advantages: (1) The GMR can achieve active locomotion in the intestinal tract so that the intestinal tract can be detected at a fixed point and the diagnostic efficiency can be improved. (2) The GMR has the function of expanding the intestinal tract so that the collapsed and folded areas of the intestinal tract can be seen to reduce missed detection. The double-layer overlapping structure of expanding arm increases the VDA of the mechanism and the contact area with the intestine, so that the robot has a certain improvement in reliability and safety to the gastrointestine. Based on the elastodynamics analysis, a structure optimization method of the expanding arm is proposed. After optimization, the overall thickness of the expanding arm is reduced by 0.4 mm, the weight of expanding arm is reduced by 31%. Then the reliability and safety of the optimized expanding mechanism are proved by experiments. The results show that this optimization method is effective and this method can also be applied to the overall structure optimization of the GMR in the future.

However, there are still some problems with the robot. The structure of the GMR in this paper can be divided into two parts, one is composed of the imaging module and the front expanding mechanism, and the other is composed of the rear expanding mechanism, the telescoping mechanism, the wireless power receiving module, and the control circuit module. There is a big difference in mass between the two parts, with the latter weight about twice as much as the former. This will decrease the locomotion efficiency of the robot. The emphasis of future work is to reduce the overall weight of the robot, optimize the weight distribution, and improve the locomotion efficiency of the robot.

## Figures and Tables

**Table 1:** Parameters of mechanical structure.

Parameter		Value
Expanding Mechanism	Size (folded)	*Φ*15 × 6.5 mm
	Size (expanded)	*Φ*49 × 6.5 mm
	VDR	3.3
	Reducer & Motor	*Φ*4 × 19.8 mm
	Reduction ratio	1024

**Table 2:** Design parameters of mechanism.

Parameter	Value
*l*_1_*/*mm	8.50
*l*_2_*/*mm	10.70
*r*_0_*/*mm	5.66
*c/*mm	1.20
*ω/*rad/s	1.70

**Table 3:** Maximum amplitude of velocity and acceleration with different expanding radius.

*r_c_* /(mm)	|Δ*v*|_max_/(mm/s)	|Δ*a*|_max_/(mm/s^2^)
7.5	4.81	587.00
8.5–9.7	0.80	158.81
22.8–24.0	0.94	189.97

**Table 4:** The maximum equivalent stress with different thickness.

*h_i_*/(mm)	*σ*_max_/(Mpa)	*σ*_[s]_/(Mpa)
1.2	428.50	864
1.0	510.94	864
0.8	917.02	864

**Table 5:** The maximum amplitude of velocity and acceleration with different expanding radius.

*r_c_* /(mm)	|Δ*v*|_max_/(mm/s)	|Δ*a*|_max_/(mm/s^2^)
7.5	10.56	1478.39
8.5–9.7	1.53	589.32
22.8–24.0	2.32	566.00
